# High expression of ZEB1 correlates with liver metastasis and poor prognosis in colorectal cancer

**DOI:** 10.3892/ol.2012.1026

**Published:** 2012-11-14

**Authors:** GUANG-JUN ZHANG, TONG ZHOU, HONG-PENG TIAN, ZUO-LIANG LIU, SHU-SEN XIA

**Affiliations:** The First Department of General Surgery, The Affiliated Hospital of North Sichuan Medical College, Nanchong, Sichuan 637000, P.R. China

**Keywords:** zinc finger e-box binding homeobox 1, colorectal cancer, prognostic factor

## Abstract

Zinc finger E-box binding homeobox 1 (ZEB1) has been shown to promote invasion and metastasis in several types of human cancer and to have a prognostic role in certain cancers. However, the clinical significance of ZEB1 in colorectal cancer (CRC) has not been sufficiently investigated. This study aimed to address this issue. In this study, we compared the expression of ZEB1 between CRC tissues and normal adjacent mucosa using quantitative real-time RT-PCR. The association of ZEB1 expression with clinicopathological characteristics was analyzed by appropriate statistical analyses. Kaplan-Meier analysis and Cox proportional hazards regression models were used to investigate the association of ZEB1 expression with survival of patients. The results showed that the relative expression levels of ZEB1 were significantly higher in CRC tissues compared to the normal adjacent mucosa and higher expression of ZEB1 correlated with liver metastasis. Kaplan-Meier analysis indicated that patients with high ZEB1 had a poor overall survival. Moreover, the multivariate analysis showed that high expression of ZEB1 was an independent predictor of overall survival. Our data indicate the potential of ZEB1 as a novel prognostic biomarker for CRC.

## Introduction

Colorectal cancer (CRC) is the third most common cancer worldwide with an estimated one million new cases and half a million mortalities every year (1). A significant number of patients with colorectal carcinoma who undergo apparently curative surgery develop local recurrence or distant metastasis, leading to shorter survival (2). For this reason, the identification of factors that accurately predict prognosis in CRC is urgently required. A deeper insight into the carcinogenesis and the factors correlated with the aggressiveness of CRC may be necessary for this requirement.

Several previous studies have revealed that epithelial-mesenchymal transition (EMT) plays a crucial role in the progression and aggressiveness of CRC (3,4). Several genes, called EMT-inducing or master EMT genes, are essential to the EMT process (5,6). Zinc finger E-box binding homeobox 1 (ZEB1) encodes a transcription factor that plays a key role in cancer progression by regulating EMT in breast, prostate, ovarian and colorectal cancer (7–11).

A previous study showed that ZEB1 was overexpressed in various CRC cell lines and crucial for the metastasis of CRC cells (12). Recently, ZEB1 was also found to be a crucial EMT inducer in human CRC and suppresses the expression of basement membrane components (13). Notably, EMT-linked loss of basement membranes indicates metastasis and poor survival in CRC (7,14).

Although a growing number of studies have demonstrated the function of ZEB1 in experimental systems, no reports have shown any clinical significance associated with ZEB1 expression in CRC. The aim of this study was therefore to clarify the clinical significance of ZEB1 expression in CRC.

## Materials and methods

### Patients and tissue samples

After obtaining adequate informed consent, surgical specimens of cancer tissue and adjacent normal mucosa were obtained from 92 patients with primary CRC who underwent surgery without preoperative treatment at the First Department of General Surgery, the Affiliated Hospital of North Sichuan Medical College, China, between January 2005 and May 2008. All tissue samples were immediately frozen in liquid nitrogen and stored at −80°C until the extraction of RNA. Clinicopathological information, including age, gender, tumor size, histological type, depth of invasion, lymph node metastasis, location, lymphatic invasion and liver metastasis, was available for all patients. The study was approved by the medical ethics committee of North Sichuan Medical College.

### Quantitative real-time reverse transcription-PCR

Total RNA was extracted from tumor tissue and adjacent normal mucosa by homogenizing tissue in TRIzol reagent (Invitrogen, Carlsbad, CA, USA), according to the manufacturer’s instructions. cDNA was synthesized using an iScript cDNA Synthesis kit (Bio-Rad Laboratories, Hercules, CA, USA). Quantitative real-time PCR was performed with an iQ SYBR-Green Supermix (Bio-Rad). All reactions were run in triplicate on the iCycler IQ multi-color Detection System (Bio-Rad). The amplification profile was denatured at 95°C for 10 min, followed by 50 cycles of denaturation at 95°C for 30 sec, annealing at 60°C for 30 sec and extension at 72°C for 1 min. The primers for ZEB1 (226 bp) were 5′-AGCAGTGAAAGAGAAGGGAATGC-3′ (sense) and 5′-GGTCCTCTTCAGGTGCCTCAG-3′ (antisense). The primers for β-actin (171 bp) were 5′-AGT TGCGTTACACCCTTTCTTGAC-3′ (sense) and 5′-GCTCGC TCCAACCGACTGC-3′ (antisense). The comparative cycle threshold (CT) method was applied to quantify the expression levels of miRNAs. The relative amount of ZEB1 was calculated using the equation 2^−ΔC^_T_ where ΔC_T_ = (C_T ZEB1_ − C_T_ β-actin).

### Statistical analysis

The gene expression levels in CRC were compared with those in normal adjacent mucosa with the use of the Wilcoxon test. The correlations between the gene expression levels and potential explanatory variables, including age, gender, tumor size, histological type, depth of invasion, lymph node metastasis, location, lymphatic invasion and liver metastasis, were evaluated with the Chi-square test. The postoperative survival rate was analyzed with the Kaplan-Meier method, and differences in survival rates were assessed with the log-rank test. A Cox proportional hazards model was used for multivariate analysis. All the statistical analyses were performed using SPSS 16.0 software (SPSS, Chicago, IL, USA). Two-sided P-values were calculated, and P<0.05 was considered to indicate a statistically significant result.

## Results

### Comparison of ZEB1 expression between CRC tissue and normal adjacent mucosa

**Figure 1.**
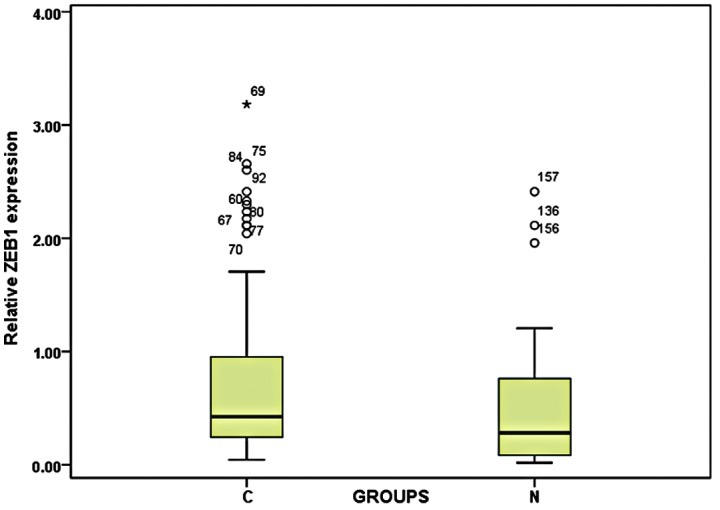
Comparison of zinc finger E-box binding homeobox 1 (ZEB1) expression levels between colorectal cancer tissue (C) and adjacent normal mucosa (N). P-value was calculated by the Wilcoxon test.

By real-time quantitative RT-PCR, we found that ZEB1 expression levels were significantly higher in cancer tissues from patients with CRC (0.843±0.693) than in the normal adjacent mucosa (0.586±0.488; P=0.003; Fig. 1).

### Correlation between ZEB1 gene expression and clinicopatho-logical features of CRC

The expression levels of ZEB1 were categorized as low or high in relation to the median value. The ZEB1 expression level was not correlated with age, gender, tumor size, histological type, depth of invasion, tumor location, lymph node metastasis or lymphatic invasion. However, the ZEB1 expression level was correlated with liver metastasis (P=0.043; Table I).

### Correlation between ZEB1 gene expression levels and survival

**Figure 2.**
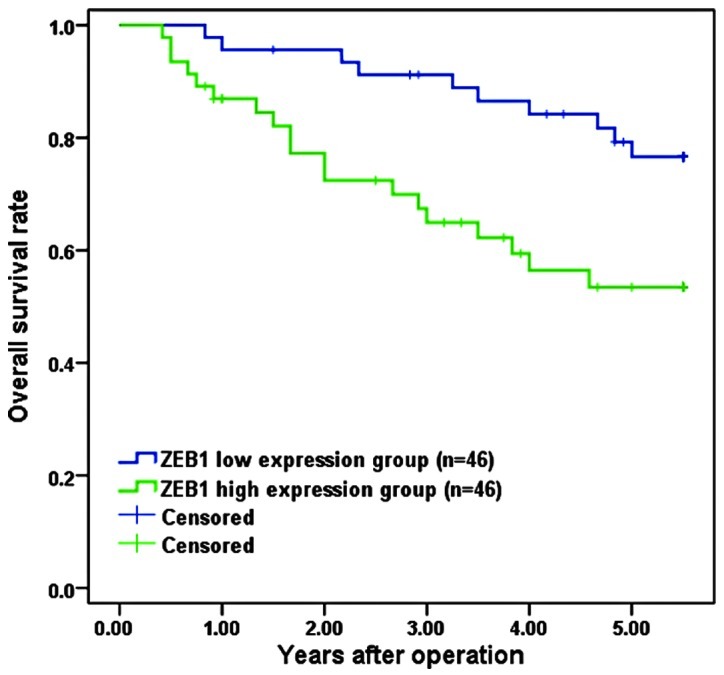
Kaplan-Meier survival curves of patients with colorectal cancer based on zinc finger E-box binding homeobox 1 (ZEB1) expression status. Patients in the high expression group (green line) had significantly poorer prognosis than those in the low expression group (blue line; P= 0.010, log-rank test).

Overall survival curves were plotted according to ZEB1 mRNA expression level by the Kaplan-Meier method. In the study group as a whole (92 patients), the overall survival rate was significantly lower in the patients with high ZEB1 mRNA expression than in those with low expression (P=0.010; Fig. 2).

### Prognostic factors of CRC

Univariate analysis with Cox proportional hazards model identified seven prognostic factors: histological type, tumor size, depth of invasion, lymph node invasion, lymphatic invasion, liver metastasis and ZEB1 expression. The other clinicopathological features, such as age, gender and location, were not statistically significant prognostic factors (Table II). A multivariate analysis of the prognosis factors with a Cox proportional hazards model confirmed that high ZEB1 expression was a significant independent predictor of poor survival in CRC (Table III).

## Discussion

Increasing evidence indicates that aberrant activation of EMT plays a key role in tumor cell invasion and metastasis. EMT allows the detachment of cells from each other and increases cell mobility, both of which are necessary for tumor cell dissemination. A hallmark for EMT is the loss of the cell adhesion molecule E-cadherin. Several transcription factors have been described as key inducers of EMT, including members of the Snail superfamily (Snail1 and Snail2), the basic helix-loop-helix (bHLH) family (TCF3 and TWIST) and the two zinc-finger E-box-binding homeobox (ZEB) factors (ZEB1 and ZEB2) (15,16).

Previous data indicate that ZEB1 has emerged as a key player in cancer progression (8,12,17,18). Aberrant expression of ZEB1 in endometrial cancers, gastric cancer and hepatocellular carcinoma has been associated with aggressive disease, poor differentiation, development of metastases and poor clinical prognosis (19–21). However, ZEB1 expression in CRC tissues and its correlation with the clinical pathology of CRC are rarely reported.

The overexpression of ZEB1 has been observed in prostate cancer, gastric cancer, osteosarcoma and hepatocellular carcinoma, suggesting an important role in tumorigenesis (11,20–22). A previous study also showed that ZEB1 was overexpressed in various CRC cell lines (12). In this study, we confirmed that ZEB1 gene expression was higher in CRC tissue than in adjacent normal mucosa, consistent with the results of previous studies.

A previous study demonstrated that high ZEB1 expression in hepatocellular carcinoma was correlated with advanced TNM stage, tumor size, intrahepatic metastasis and vascular invasion (21). Okugawa *et al* reported that increased ZEB1 expression in gastric cancer was not clearly associated with histological type, tumor size, lymph node metastasis and hepatic metastasis, and elevated ZEB1 expression was shown to be significantly correlated with peritoneal dissemination (20). However, the correlation between ZEB1 expression level and clinicopathological behavior of CRC is unclear. In the present study, ZEB1 expression was shown to be associated only with liver metastases, suggesting that ZEB1 is involved in the carcinogenesis, development, progression and metastasis of CRC. The inconsistent results may be due to different roles of ZEB1 in different tumors.

More importantly, we proved that ZEB1 expression was significantly associated with overall survival of patients with CRC. In support of this, the Kaplan-Meier analysis of overall survival showed that patients whose tumors had higher ZEB1 expression tend to have a significantly worse overall survival, indicating that a high ZEB1 level is a marker of poor prognosis for patients with CRC. Moreover, Cox proportional hazards model showed that ZEB1 was a marker of poor overall survival independent of the known clinical prognostic indicators such as lymph node and liver metastases. Therefore, it could constitute a molecular prognostic marker for these patients, identifying those patients who are more likely to have a higher risk of mortality; thus, good candidates to receive more aggressive treatment.

The precise molecular mechanisms behind the altered expression of ZEB1 in CRC are unclear. To the best of our knowledge, this is the first study to describe the significance of ZEB1 to liver metastasis and prognosis of CRC patients. ZEB1, which is also known as TCF8 or ΔEF1, is a member of the zinc finger family (22). It had been found that ZEB1 inhibits the expression of multiple genes in a variety of cell lines. In hematopoietic cells, ZEB1 negatively regulated the expression levelss of IL-2, CD4, the heavy chain of immunoglobulin μ and GATA-3 (23–25). In mesenchymal cells, ZEB1 inhibited the expression of the p73 gene (26). Recent studies found that ZEB1 also inhibited the expression of epithelial cadherin (E-cadherin), and thus plays an important role in the carcinogenesis, progression, invasion and metastasis of a variety of tumors (27,28). Putzke *et al* reported that the overexpression of ZEB-1 in prostate cancer cells inhibited the expression of E-cadherin protein, thereby promoting the invasion and metastasis of prostate cancer cells (27). The overexpression of ZEB1 was also found to promote the invasion and metastasis of lung cancer cells (29). In addition, ZEB1 promotes the epithelialmesenchymal transformation of liver cancer cells (30). Taken together, these studies may explain why ZEB1 overexpression is associated with poor prognosis of CRC patients. Additional studies to investigate the molecular mechanisms of both the cause and effects of altered expression of ZEB1 in the development and/or progression of CRC are essential.

In conclusion, we have shown that ZEB1 expression was increased in clinical CRC specimens and associated with liver metastasis and poor prognosis. This study further demonstrated that ZEB1 was an independent prognostic factor of patients with CRC. These findings suggest that ZEB1 may be a potential diagnostic and therapeutic target in patients with CRC.

## Figures and Tables

**Table I.** Correlation between ZEB1 expression and clinico-pathological parameters in 92 colorectal cancer patients.

Variable	ZEB1 expression		P-value
	Low (n=46)	High (n=46)	
Age (years)	62.8±14.6	61.1±11.7	0.519
Gender			0.209
Male	28	22	
Female	18	24	
Tumor size (cm)			0.288
≤5	30	25	
>5	16	21	
Histological type			0.529
Well, moderate	27	24	
Poor, mucinous	19	22	
Depth of invasion			0.674
T1,T2	21	19	
T3,T4	25	27	
Location			0.669
Colon	19	17	
Rectum	27	29	
Lymph node metastasis			0.058
Absent	24	15	
Present	22	31	
Lymph node invasion			0.084
Absent	33	25	
Present	13	21	
Liver metastasis			0.043^a^
Absent	40	32	
Present	6	14	

ZEB1, zinc finger E-box binding homeobox 1; n, number of patients; well, well-differentiated; moderate, moderately differentiated; poor, poorly differentiated.

**Table II.** Univariate analysis of clinicopathological factors for overall survival.

Variable	n	Hazard ratio	95% CI	P-value
Age (years)				
≤65	40	1		
>65	52	1.455	0.701–3.019	0.314
Gender				
Male	50	1		
Female	42	1.274	0.608–2.669	0.521
Tumor size (cm)				
≤5	55	1		
>5	37	3.837	1.771–8.312	0.001^a^
Histological type				
Well, moderate	51	1		
Poor, mucinous	41	2.401	1.141–5.052	0.021^a^
Depth of invasion				
T1, T2	40	1		
T3, T4	52	3.247	1.383–7.625	0.007^a^
Location				
Colon	36	1		
Rectum	56	1.674	0.807–3.473	0.167
Lymph node metastasis				
Absent	39	1		
Present	53	8.956	2.704–29.663	<0.001^a^
Lymph node invasion				
Absent	58	1		
Present	34	3.820	1.797–8.121	<0.001^a^
Liver metastasis				
Absent	72	1		
Present	20	15.427	6.342–37.528	<0.001
ZEB1				
Low	46	1		
High	46	2.646	1.226–5.710	0.013^a^

n, number of patients; CI, confidence interval; ZEB1, zinc finger E-box binding homeobox 1; well, well-differentiated; moderate, moderately differentiated; poor, poorly differentiated.

a P<0.05.

**Table III.** Multivariate analysis of clinicopathological factors for overall survival.

Variable	Hazard ratio	95% CI	P-value
Tumor size (>5 cm/≤5 cm)	1.779	0.736–5.044	0.182
Histological type (poor, muc/well, mod)	1.852	0.841–4.079	0.126
Depth of invasion (T3, T4/T1, T2)	1.548	0.620–3.864	0.349
Lymph node metastasis (present/absent)	6.165	1.733–21.926	0.005^a^
Lymphatic invasion (present/absent)	2.001	0.788–5.080	0.144
Liver metastasis (present/absent)	4.816	1.794–12.929	0.002^a^
ZEB1 (high/low)	2.237	1.008–4.968	0.048^a^

CI, confidence interval; well, well-differentiated; mod, moderately differentiated; poor, poorly differentiated; muc, mucinous; ZEB1, zinc finger E-box binding homeobox 1.

a P<0.05.
